# Cost-Effectiveness of Inter-Professional Collaboration to Reduce Hospitalisations in Nursing Home Residents: Results from the German Interprof ACT Trial

**DOI:** 10.5334/ijic.7001

**Published:** 2023-04-19

**Authors:** Louisa-Kristin Muntendorf, Katrin Balzer, Tim Friede, Eva Hummers, Hans-Helmut König, Christiane Müller, Martin Scherer, Linda Steyer, Britta Tetzlaff, Sebastian Pfeiffer, Alexander Konnopka, on behalf of the interprof ACT Consortium

**Affiliations:** 1 University Medical Center Hamburg-Eppendorf, Institute of Health Services Research and Health Economics, Martinistraße 52, D-20246 Hamburg, Germany; 2 Institute for Social Medicine and Epidemiology, Nursing Research Unit, University of Lübeck, Ratzeburger Allee 160, Haus 50, D-23538 Lübeck, Germany; 3 Department of Medical Statistics, University Medical Center Göttingen, Humboldtallee 32, D-37073 Göttingen, Germany; 4 Department of General Practice, University Medical Center Göttingen, Humboldtallee 38, D-37073 Göttingen, Germany; 5 Department of General Practice and Primary Care, University Medical Center Hamburg-Eppendorf, Martinistraße 52, D-20246 Hamburg, Germany; 6 Department Psychology, MSH Medical School Hamburg, Am Kaiserkai 1, D-20457 Hamburg, Germany

**Keywords:** integrated care, cost-effectiveness, nursing homes, physician-nurse, inter-professional relations, randomised controlled trial

## Abstract

**Background:**

The German multi-centre cluster-randomised controlled trial *interprof ACT* investigated interventions to increase inter-professional collaboration between nursing home (NH) staff and local general practitioners to reduce hospitalisations and improve nursing homes residents’ (NHRs) quality of life. The trial was funded by the German Health Care Innovation Fund.

**Methods:**

Cost-effectiveness of *interprof ACT* interventions was evaluated and compared to current standard of care (SOC) over 12 months, including 622 NHRs in 34 NHs in Germany. Multiplying resource use of healthcare services with German-specific unit costs generated costs. Health outcome was measured in quality-adjusted life-years QALYs), utility by multiplying EQ-5D-5L values with German-specific utility weights. Incremental cost-effectiveness analysis used an intention-to-treat approach and scenario analyses (SAs). Net-benefit-regression and cost-effectiveness acceptability curves addressed uncertainty. A German healthcare insurance perspective was assumed.

**Results:**

Base case results showed non-significant cost savings of 851.88€ and non-significant QALY loss of –0,056.

**Discussion:**

Dependency levels at baseline were non-significantly higher in IG compared to control group (CG). Lack of baseline costing data eliminated possibility to evaluate changes in costs due to the *interprof ACT* measures for both groups.

**Conclusion:**

*Interprof ACT* interventions are not cost-effective compared to current SOC.

## Introduction

With progression in age people often become more likely to require assistance in their daily life. To provide these patients with the best suitable care provision, admissions to nursing homes (NH) pose a possible solution. In 2019, nearly 20% of all German patients with a need of care lived in NH [1]. In Germany, first line medical assistance for nursing home residents (NHR) is provided by the general practitioner, receiving financial compensation based on the Doctors’ Fee Scale within the Statutory Health Insurance Scheme (Einheitlicher Bewertungsmaßstab; EBM) [2].

Acute changes of a NHRs’ medical condition, which require immediate hospital admission, are often traumatizing and bear additional health risks for the NHR, such as pressure ulcers or nosocomial diseases [3]. In Germany, it is assumed that 30–60% of all NHRs are at least once a year admitted to a hospital, with 40% of hospital admission being preventable according to international data [2,4,5,6,7]. Reasons for hospitalisation include but are not limited to falls, fractures, cardiovascular diseases, and respiratory tract infections [3,7,8]. Unplanned hospitalisations are often due to the nursing and/or medical staff in charge being unable to assess the severity of the acute medical condition or the lack of a concise plan of action in the case of severe health deterioration of a NHR [9,10]. Studies suggest a steady flow of communication between general practitioners (GPs) and nursing staff and a fixed contact person could potentially reduce the number of unplanned hospitalisations [10]. Strengthening the inter-professional collaboration between GPs and nursing staff could therefore not only reduce hospital admission related co-morbidities of NHRs but also serve as a tool to monitor and/or foresee severe health deteriorations of the NHR at an earlier stage. Averted hospitalisations not only have a positive impact on the quality of life of the NHR, but also save the costs of potential malpractices resulting from inefficiencies in the healthcare system, such as over treatment.

The German cluster-randomised controlled trial *interprof ACT* evaluated whether a closer collaboration of the NH staff and the NH’ GP in terms of information and communication flow, as well as a direct communication of (future) medical treatment decisions could reduce the cumulative incidence of hospitalisations within 12 months measured through reduced costs and increase the NHR’s quality of life compared to the current standard of care. Although the trial showed no significant reduction in hospitalisations in the IG, comparing additional gains in quality of life with cost savings through closer collaboration of GPs and NH staff could still yield the intervention to be cost-effective.

The following economic evaluation of the German *interprof ACT* trial seeks to determine whether the intervention is cost-effective using quality-adjusted life-years (QALY) as effectiveness measure. The study was conducted from a healthcare insurance perspective.

This project has received ethical approval from the ethics committee of the Medical Faculty, Georg-August-University Göttingen, Germany (no. 31/7/17) as well as ethical approval from the ethics committee of the University of Lübeck (no.18–051), and the ethics committee of the University Medical Center Hamburg-Eppendorf (no. MC-304/17). The *interprof ACT trial* is funded by a public organism after competitive peer review (German Innovation Fund. Proposal ID VF1_2016-079).

## Methods

### Intervention and Study Population

The multicentre, cluster-randomised controlled trial *interprof ACT* was conducted between 2018 and 2020 in 34 NHs in Germany in the cities and catchment areas of Hamburg (n = 16), Lübeck (n = 10) and Göttingen (n = 8)., and included 622 NHRs. NHs were randomised pairwise 1:1 to IG and CG, and followed-up at 12 months after randomisation. Patient cluster consisted of on average 19 NHRs in each NH. The overall number of patients in the trial was 672, out which 50 patients were excluded for this cost-effectiveness analysis due to missing data. The remaining 622 patients were distributed evenly among both groups with 311 patients in IG and 311 in CG with an average age of 82 years in both groups. Further information on baseline characteristics and randomisation of patient groups can be found elsewhere [11]. The CG received standard care with brief information on medical and nursing care. In the IG, the appropriate *interprof* ACT measures were jointly selected by NH and general practitioner, implemented, and applied over the 12-month trial period.

*Interprof ACT* consists of six components, each supported by specific strategies, which were implemented over 12 months (Table 1) [12]. The implementation of the *interprof ACT* strategies did not interfere with any already ongoing medical treatment plan for patients in the NHs selected for the *interprof ACT* trial. For each participating NHs, a designated *interprof ACT* agent (IPAV) was selected to facilitate and oversee the implementation of the interventions, as well as the materials to enforce the *interprof ACT* measures, such as name badges. Supervision and support of IPAVs was given by the study team over telephone, e-mail, and face-to-face meetings.

**Table 1:** Interprof ACT measures.


INTERPROF ACT COMPONENTS	INTERPROF ACT IMPLEMENTATION STRATEGIES

Use of name badges worn by GPs and nurses during the GPs’ visits	Designated *interprof ACT* agents (IPAVs) in nursing homes

Appointment of a contact person in nursing home and GP’s practices	Involvement of NHRs’ GPs into the implementation process

Mandatory availability via phone and fax	In-house kick-off meeting involving all parties

Standardized procedures for GPs’ home visits	Involvement of NHRs into the implementation process

Support in assigning administration of prescribed medication	Support for implementation and materials for communication

Meetings for shared goal setting	


### Cost and Utility

In order to carry out the economic evaluation, the use of relevant health services by the NHR was first assessed with a questionnaire among the respective caregivers based on the NHRs medical record. The questionnaire was based on the FIMA questionnaire, from which relevant items were selected. The FIMA questionnaire is a generic questionnaire, which collects the health related resource use for older population groups [13]. Further relevant items were identified and added after receiving approval from the *interprof ACT* consortium. Resource use of medical services by NHR was collected for retrospective periods of 6 months at T1 and T2 from each NHRs medical file. The EQ-5D-5L was collected at T0 and T2.

After obtaining the dataset, average German unit costs were used to calculate costs of care for both groups based on the use of medical services (Table 2) [14,15]. If unit costs (UCs) were unavailable from the literature, a UC was calculated by the authors according to the PECUNIA methodology using the PECUNIA Resource Use Measurement Template and PECUNIA Reference Unit Cost Compendium [16,17,18]. If necessary, UCs were inflated to the year 2020 using the German Consumer Price Index [19,20]. Responses on the EQ-5D-5L were used to calculate quality-adjusted life-years (QALYs) using published utility weights for Germany [21].

**Table 2:** Unit cost of medical services in Germany used in cost-effectiveness analysis.


MEDICAL SERVICE	UNIT COST IN € (ORIGINAL YEAR)	UNIT COST IN € (2020)	REFERENCE

General practitioner (per visit)	20.06 (2011)	22.29	[14]

Gynaecologist (per visit)	30.13 (2011)	33.48	[14]

Orthopaedist (per visit)	25.42 (2011)	28.25	[14]

Internist (per visit)	65.44 (2011)	72.73	[14]

Ophthalmologist (per visit)	34.34 (2011)	38.16	[14]

Dermatologist (per visit)	18.89 (2011)	20.99	[14]

ENT specialist (per visit)	26.40 (2011)	29.34	[14]

Surgeons (per visit)	43.39 (2011)	48.22	[14]

Urologists (per visit)	24.70 (2011)	27.45	[14]

Neurologists (per visit)	44.72 (2011)	49.70	[14]

Psychotherapists (per visit)	78.08 (2011)	86.77	[14]

Other groups of doctors (per visit)			

Radiologist (per visit)	56.04 (2011)	62.28	[14]

Dentist (per visit)	55.87 (2011)	62.09	[14]

Podiatrist (per visit)	27.51 (2011)	30.57	[14]

Occupational therapist (per visit)	37.51 (2011)	41.69	[14]

Speech therapist (per visit)	38.59 (2011)	42.89	[14]

Physiotherapist (per visit)	16.42 (2011)	18.25	[14]

Hospital (per day)			

Intensive care unit	1,337.72 (2011)	1,486.67	[14]

Full inpatient	575.9 (2011)	640.02	[14]

Partial inpatient	374.33 (2011)	416.01	[14]

Psychiatry (per day)			

Inpatient	339.71 (2011)	377.53	[14]

Partial inpatient	220.81 (2011)	245.40	[14]

Therapeutic products			

Aids for compression therapy	140.66 (2011)	156.32	[14]

Incontinence aids	346.43 (2011)	385.00	[14]

Inhalation and respiratory therapy devices	967.75 (2011)	1,075.50	[14]

Inpatient rehab			

without addiction (per Day)	111.10 (2011)	123.47	[15]

Art therapy (per visit)	68.40 (2014)	72.73	[15]

Music therapy (per visit)	56.60 (2014)	60.18	[15]

Movement therapy (per visit)	58.33 (2014)	62.02	[15]

Sex therapist (per visit)	See Psychotherapists	See Psychotherapists	[14]

Alternative/Holistic practitioner (per visit)	–	106.08	*

Association of Statutory Health Insurance Physicians Emergency Service (per visit)	–	182.71	*

Ambulance (per visit)	–	1,050.04	*

Emergency room (per visit)	–	133.31	*

Individual talk therapy (per visit)	See Psychotherapists	See Psychotherapists	[14]

Talk therapy group (per visit)	See Psychotherapists	See Psychotherapists	[14]

Wound therapist/Compression Therapist (per visit)	–	156.32	* +


* Own Calculation according to PECUNIA Ressource Use Measurement Template and PECUNIA Reference Unit Cost Compendium [16,17,18]. + Costs wound therapist: There is currently no DRG for wound care. The billing of treatment costs for chronic wounds was changed by the G-BA in November 2020. However, as of 09.05.2022, there is still no DRG according to which billing can take place. Billing as compression therapy is possible.

### Analysis

Cost-effectiveness analysis was performed, in which QALYs were the measure of effects. In the baseline scenario we performed an intention-to-treat analysis, which compared treatment groups and included every NHR randomised according to the *interprof ACT* measures. This is the recommended method in superiority trials to avoid any bias [22]. For missing observations in individual cost categories and/or EQ-5D-5L scores, the “last observation carried forward” (LOCF) method was used.

To calculate QALYs, health-related quality of life (HRQL) index scores from the EQ-5D-5L were firstly collected, which is a popular generic indirect preference-based measure of HRQL [23]. The EQ-5D-5L uses a descriptive system with five dimensions of health, each with five response options describing levels of impairment from “no problems” (level 1) to “extreme problems” or “unable to do” (level 5). If NHRs were unable to complete the EQ-5D-5L due to their medical conditions, such as dementia or other cognitive impairments, NH staff assisted in recalling the HRQL based on the NHRs specific medical documentation.

This individual QOL data collected at T0 and T2 is weighted by a value set, which reflects the German general public’s health preferences, and is then used to generate the quality of life weights (“utilities”) required to estimate QALYs [24].

QALYs for both groups were estimated by first calculating NHR’s individual QALYs over the trial period of 12 months using the sum of the utility values at T0 and T2 (12 months) divided by two, which corresponds to the linear interpolation of utility values between T0 und T2. The effect of the intervention, measured in QALYs, corresponds to the difference in mean QALYs ( \overline E) between the IG and the CG [25]. To calculate mean cost per group ( \overline C), individual health-related costs of NHRs was assessed and aggregated to individual overall costs for IG and CG [26].

Incremental cost-effectiveness ratio (ICER) was calculated as the main outcome measure, which is defined as the ratio of the differences in mean cost \overline C and mean effects \overline E between IG and CG:

{\rm{ICER}} = \frac{{{{{\rm{\bar C}}}_{{\rm{interpr\ of\ ACT\ intervention}}}} - {{{\rm{\bar C}}}_{{\rm{Control\ Group}}}}}}{{{{{\rm{\bar E}}}_{{\rm{interpr\ of}}{\rm{\ ACT\ intervention}}}} - {{{\rm{\bar E}}}_{{\rm{Control\ Group}}}}}} = \frac{{\Delta {\rm{\bar C}}}}{{\Delta {\rm{\bar E}}}}

[27].

In addition, three scenario analyses were performed:

In scenario analysis (SA) 1, the QALY utility weight for NHRs with missing EQ-5D-5L values at T2 was set to “0” at the time of death. In SA2, a complete-case analysis was performed, which only includes participants who had no missing data on the variables of interest. Participants with any missing data are excluded [28]. In SA3, a subgroup analysis was performed for NHRs with Care Degree (CD) 4 and 5. In Germany, patients receive statutory long-term care benefits based on five CDs ranging from 0 (no need for care) to 5 (high need for care) [29]. The level of benefits patients receive varies based on their CD as well as patients’ living arrangements and increases with increasing CD. In all scenarios, costs and QALYs were first compared between treatment groups and the incremental cost-effectiveness ratio (ICER) was calculated as an outcome measure. To determine the significance level for cost and QALY differences, regression analyses were performed with age, sex, level of care, number of previous medical conditions, and treatment group as independent variables. A generalized linear model (GLM) with Gamma distribution and log-link function was used for the analysis of costs, and OLS regression was used for the analysis of QALYs [28].

### Uncertainty Assessment

A net benefit regression was performed to estimate uncertainty of the results of the cost-effectiveness analysis [30]. For this purpose, individual net monetary benefits (NMB) were calculated for different willingness-to-pay (WTP) threshold ranging from 0€/QALY to 250,000€/QALY. Net monetary benefit (NMB) is a summary statistic that represents the value of an intervention in monetary terms when a WTP for a unit of benefit (here QALY) is known [26]. NMB is calculated as follows:

\begin{array}{l}
{\rm{NMB\ =\ (Incremental\ Benefit(}}\Delta \overline {\rm{E}} {\rm{)}} \times \\
\;\;\;\;\;\;\;\;\;{\rm{WTP\ Threshold\ (}}0\euro - 250,000\euro )) - {\rm{Incremental\ Costs\ (}}\Delta {\rm{\bar C)}}{\rm{.}}
\end{array}

**Figure 1:**
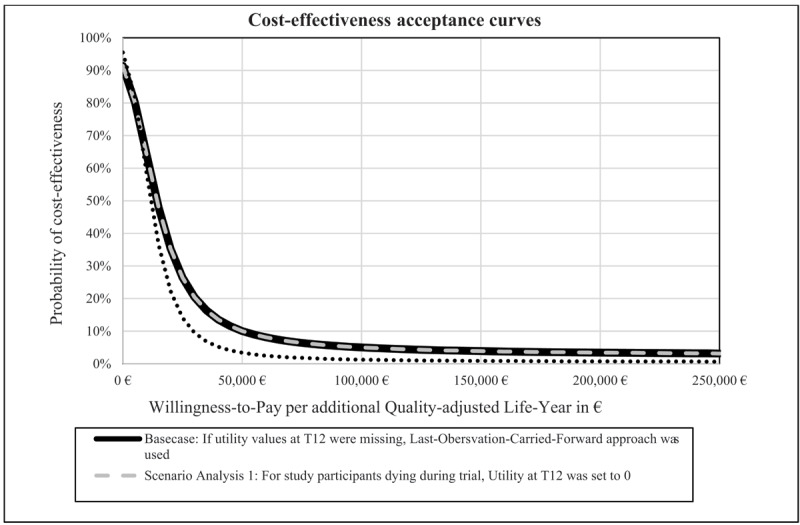
Cost-effectiveness acceptability curves.

The results of the NMB for each WTP threshold were used to form cost-effectiveness acceptability curves (CEACs) for the base case and the following scenario analyses to evaluate the probability of cost-effectiveness of the intervention (Figure 1).

All analyses were conducted from a social insurance perspective. Because all study participants were NHRs and therefore, by definition, no longer employed, no effects of the intervention on indirect costs were expected; all other costs analysed were borne by healthcare insurance. Therefore, an additional analysis from a societal perspective was not useful. Analysis was carried out with IMB SPSS Statistics 27 (Armonk, NY, USA).

## Results

### Base Case

In the base case (BC) analysis, the IG showed lower costs compared to CG (4,738.79€ vs. 5,590.67€) but no improvement in QALYs (IG: 0.482; CG: 0.537), resulting in a non-significant mean cost saving of 851.88€ and a likewise non-significant mean QALY loss of –0.056QALYs (Table 3).

**Table 3:** Results of the cost-effectiveness analysis.


SCENARIO	PARAMETER	CONTROL GROUP	INTERVENTION GROUP	DELTA	p-VALUE	ICER

**Basecase**						

Number of patients		311	311			

	Cost	5,590.67€	4,738.79€	–851.88€	0.172	15,288.83€

	QALY	0.537	0.482	–0.056	0.047	

**Scenario 1**						

Number of patients		311	311			

	Cost	5,590.67€	4,738.79€	–851.88€	0.172	15,288.83€

	QALY	0.537	0.482	–0.056	0.047	

**Scenario 2**						

Number of patients		223	248			

	Cost	5,292.12€	4,370.98€	–921.14€	0.9	10,953.77€

	QALY	0.552	0.468	–0.084	0.009	


Mean total intervention costs, which were added in the IG to the mean total costs, amounted to 425.94€. Inpatient care and rehabilitation accounted for the largest proportion of the mean total costs in both groups (IG: 1,798,51€; CG: 2,202.7€), followed by medication including medical supplies (IG: 994€; CG: 979€) and outpatient physician services (IG: 856€; CG: 981€).

The ICER was 15,288.83€/QALY. As the intervention was simultaneously worse (fewer QALYs) and cheaper, the ICER can be interpreted as an ICER of the CG: the lower the ICER, the more cost-effective the control condition. This is reflected in the cost-effectiveness acceptability curve (CEAC) which shows that at a starting threshold of 0€ per QALY, there was a 91.4% probability that the intervention was cost-effective, which decreased continuously to a probability of 3.2% as willingness to pay (WTP) increased to 250,000€ per QALY (Figure 1).

### Scenario Analyses

In SA1, where the QALY utility weight for deceased NHRs was set to “0” at the time of death, the QALY loss was reduced by 0.056 QALYs (Table 3) in the IG compared to the BC because more deaths occurred in the CG than in the IG. Similar to the BC, costs in the IG equalled costs of BC and were lower than in the CG (IG: 4,738.79€ vs. CG: 5,590.67€; IG: 0.482 QALY vs. CG: 0.537), increasing the ICER to 15,288.83€/QALY compared to the BC. The probability of cost-effectiveness, similar to BC, continuously decreases from 91,4% at a WTP threshold of 0€ to 3.15% at a WTP threshold of 250,000€.

In SA2, a complete-case analysis was performed, excluding all NHRs that did not complete the 12-month trial period and therefore reducing the number of NHRs included in the analysis. Results of SA2 showed similar results as BC and SA1, with less costs and QALYs in the IG compared to CG (IG: 4,750€ vs. CG: 4,885€; IG: 0.4938 QALYs vs. CG: 0.5271 QALYs) and an ICER of 4,045€ per QALY. The CEAC shows that the probability of cost-effectiveness continuously decreases from 76.4% at a WTP threshold of 0€ to 26.7% at a WTP threshold of 250,000€.

## Discussion

The analysed *interprof ACT* intervention to improve quality of life for NHRs through closer collaboration between GPs and NH did not prove to be cost-effective. During the 12-month follow period, quality of life did not improve significantly more in the NHRs where the *interprof ACT* measures were implemented. In combination with direct healthcare costs tending to be higher in the participants of the CG, this results in a very low probability of the intervention being cost-effective. For example, at the frequently used cost-effectiveness threshold of 50,000€/QALY, the probability of the intervention being cost-effective was only 10% if total costs and societal preferences values were used for calculating the ICER. When calculating costs and QALYs in the three different scenario analyses, the ICER was not impacted. Although it was assumed that patients with the highest care degrees 4 and 5 could profit from the *interprof ACT* intervention, due to more medical care via GP and therefore prevention of hospitalisations, data could not prove this. Despite the *interprof ACT* results, the idea of increasing inter-professional care in nursing homes to avoid hospitalisations is an important research topic in the German healthcare community. To find expedient solutions, the German Innovation Fund finances studies with different intervention components such as the German CoCare (coordinated medical care) project conducted in 2022 [31]. The aim of the CoCare project was to improve coordinated medical care in nursing homes and to optimize the interface between care and physicians within the healthcare system in order to reduce avoidable hospital admissions and patient transports. The new form of care includes joint visits by nursing home coordinators, joint training courses, primary care physicians, and, as opposed to the *interprof ACT* trial, medical specialists, the formation of teams of physicians, a joint electronic patient file, and extended accessibility to medical care. The results of the centralised cost-benefit analysis of total costs of the CoCare project revealed a significant reduction of total health care costs for the IG compared to the CG. The total costs of medical service utilization were reduced due to the intervention by per NHR and quarter in the IG compared to the KG by 231.27€ [adjusted 468.56€; p < 0.001]. Thus, an extremely advantageous cost-benefit ratio can be assumed. Similar to *interprof ACT*, the CoCare project could not detect a significant reduction of hospitalisations across patients. The results of CaCare and *interprof ACT* show that a further evaluation of services and professionals included in future study design is necessary in order to achieve a reduction of hospitalisations.

The results of the *interprof ACT* trial stand in line with results of international trials, which have also shown mixed results [32,33]. Research suggests, that NHs with high implementation rates of the new interventions achieve lower rates of all-cause hospitalisation rates compared to those with poor implementation rates [34]. A possible explanation could be the financial, as well as staff resources of the NH at baseline, which could determine the level of time and effort spent on implementing new measures.

### Possible explanations for the lack of success

Various factors could influence the results of this analysis, among which the timeframe of the study may play an important role.

Due to the COVID-19 pandemic during the last two months of the trial period, face-to-face meetings of *interprof ACT* team members, NHRs, NH staff, and GPs were difficult to facilitate. While government health requirements restricted access to NHs, its staff had limited digital devices available to communicate with the *interprof ACT* team [35,36]. Furthermore, staff shortages went hand in hand with increased demands in healthcare provision for NHRs and left little to no time for staff to complete study documentation [37].

These circumstances could explain why EQ-5D-5L data at T2 was only available for 92% of study participants. As a consequence, LOCF imputation was performed, i.e. the baseline determination of the missing NHRs EQ-5D-5L values were imputed as T2 determination [38]. Thus, chances of under- and overestimation of values is possible: underestimation will occur if the health-state of the NHR had improved over the trial period; overestimation will occur if the health-state of the NHR had worsen or died during the trial period. However, as the IG was affected more by this bias compared to the CG, the effect on the ICER could potentially be high, since the ICER is calculated by differences between the IG and CG.

Although our analysis controlled for the number of comorbidities in both IG and CG, the overall health-state of the IG was worse than of the CG at baseline, which in Germany is indicated by Care Degrees (CD). CDs are used to allocate patients to one of five levels of need, ranging from 0 (no need for care) to 5 (high need for care) [29]. The level of financial support depends on the CD as well as patients’ living arrangements. For patients living at home, monetary benefits provided by the German insurance system are lower compared to patients receiving full-time care in NHs. In our analysis, cost of NHs was not included because all trial participants resided in NHs. Baseline data showed that there were more NHRs with CD4 and CD5 in the IG than the CG. This consequently generated higher costs in the CG vs. IG. In addition, NHRs resource use was collected from medical documentation only, which could lead to an under- or overestimation of treatment costs in both groups if any NHRs treatments were not documented.

### Limitations

The *interprof ACT* trial did not collect baseline cost data of NHRs, eliminating the possibility to evaluate changes in cost in the trial period due to the intervention. As previously mentioned, higher CDs incur higher cost. Therefore, comparing baseline costs of both, CG and IG, to T2 could show if the lower costs of the IG in the baseline analysis are in fact driven by the implementation of the *interprof ACT* measures [39].

## Conclusion

The intervention of the *interprof ACT* trial to increase inter-professional collaboration between GPs and NHs in Germany is not cost-effective.

## Trial registration

ClinicalTrials.gov ID: NCT03426475, February 7, 2018.
